# Optimising the implementation of a universal web-based mental health service for Australian secondary schools: a cluster randomised controlled trial

**DOI:** 10.1186/s13034-025-00975-5

**Published:** 2025-12-26

**Authors:** Mirjana Subotic-Kerry, Andrew Mackinnon, Dervla Gallen, Simon Baker, Belinda Louise Parker, Melinda Rose Achilles, Cassandra Chakouch, Nicole Cockayne, Helen Christensen, Bridianne O’Dea

**Affiliations:** 1 Black Dog Institute, University of New South Wales, Sydney, NSW Australia; 2 Orygen, University of Melbourne, Melbourne, Australia; 3 Discipline of Psychiatry and Mental Health, University of New South Wales, Sydney, Australia; 4 Flinders Institute for Mental Health and Wellbeing, College of Education, Psychology and Social Work, Flinders University, Adelaide, Australia

**Keywords:** School, Web-based, Digital, Student, Help-seeking, Mental Health, Implementation

## Abstract

**Background:**

Secondary schools are increasingly delivering a range of mental health interventions with varied success. This trial examined the effectiveness of two implementation strategies, allocation of class time and provision of financial incentives, on the engagement of secondary students with a universal web-based mental health service, *Smooth Sailing*.

**Methods:**

A three-arm, cluster-randomised trial was conducted over 12 weeks with Grade 8 and 9 students from 20 schools in two Australian states. Schools were randomised to: (1) the standard *Smooth Sailing* service, (2) the standard service plus extra class time, or (3) the standard service plus financial incentives. The primary outcome was student engagement, measured by the number of modules accessed at 12-weeks post-baseline. Secondary outcomes included uptake, retention, help-seeking intentions for mental health problems, service satisfaction, and barriers to use.

**Results:**

A total of 20 schools consented, and 1295 students participated. Students accessed a higher number of modules in the enhanced conditions compared with the standard service, but the differences were not statistically significant (*p* = 0.14). There were no significant differences in uptake (*p* = 0.55) or retention (*p* = 0.95) between conditions. Help-seeking intentions significantly improved at 6- and 12-weeks in the standard service and class time conditions only. Common barriers to service use among students were forgetfulness and low motivation.

**Conclusions:**

Neither class time allocation nor financial incentives significantly increased student engagement, as measured by modules accessed, highlighting the challenges of optimising engagement with digital mental health services in schools and emphasising the need to consider the broader school context.

*Trial registration* Australian New Zealand Clinical Trial Registry (ACTRN12621000225819) and Universal Trial Number (U1111-1265-7440).

**Supplementary Information:**

The online version contains supplementary material available at 10.1186/s13034-025-00975-5.

## Introduction

Digital mental health interventions are increasingly being delivered in schools to improve access to mental health resources and support student well-being [1]. Secondary schools, particularly in Australia, the United Kingdom and the United States, have become key settings for delivering mental health interventions, with students utilising school-based services as often as they do in traditional healthcare settings [2–5]. However, like other service sectors, schools face significant implementation challenges that limit the uptake, integration, and sustainability of evidence-based interventions. While schools are diverse and dynamic settings, few studies have gone beyond the confines of clinical trials to examine the real-world" translation of these interventions [6]. Consequently, many school-based mental health interventions have failed to achieve scalability. Evaluating the effectiveness of implementation strategies to increase service engagement among students may help to extend the reach and impact of these interventions.

School-based mental health programs and services vary in intervention type, delivery method, levels of support, and degree of youth involvement. Universal interventions, which target all students regardless of their mental health status or risk, offer several advantages over targeted approaches. They increase protective factors, reduce stigma [7, 8], and align with whole-of-school well-being policies, making them a preferred option for schools [9, 10]. Universal, digital mental health programs have demonstrated benefits for young people's emotional outcomes including improved resilience, coping skills, and self-efficacy [11]. Small improvements in help-seeking intentions [12], and modest effects on depression and anxiety outcomes have also been reported [13, 14]. However, low engagement and high dropout rates have been a persistent issue in past interventions [15], and significant challenges remain in optimising student engagement and adherence to these programs [1, 16, 17].

Improving student engagement with digital mental health programs is crucial for ensuring adequate exposure to the therapeutic content within school-based interventions. A systematic review and meta-analysis found significant associations, albeit modest, between engagement and improved mental health outcomes (effect sizes: r = 0.23 to Hedges’ g =  − 0.40), with module completions emerging as a key engagement metric [18]. Similarly, Donkin et al. [19], found that improvements in individuals’ mental health outcomes were linked to the number of modules accessed, but not to other engagement metrics such as time spent or logins. Strategies such as allocating class time and providing teacher support can significantly enhance completion rates when compared to self-directed completions. For example, school-based interventions supervised by teachers have shown higher completion rates than those accessed independently by students in the community [20]. Additionally, human guidance, including feedback and support, has been shown to increase completion rates in web-based interventions for depression and anxiety in adults [21].

Emerging evidence also suggests that financial incentives may enhance engagement with digital programs. A recent review found that app-based programs offering monetary incentives achieved higher retention rates compared to those without incentives [22, 23]. Similarly, in a randomised controlled trial, adolescents were more likely to complete an online, self-guided, single-session mental health intervention when paid, compared to those in an unpaid, naturalistic setting [24]. Small financial incentives have also been shown to improve adherence to a web-based intervention promoting physical activity in adults [25], as well as increase survey response rates and patient compliance [26].

### Implementation of the smooth sailing service

**Fig. 1:**
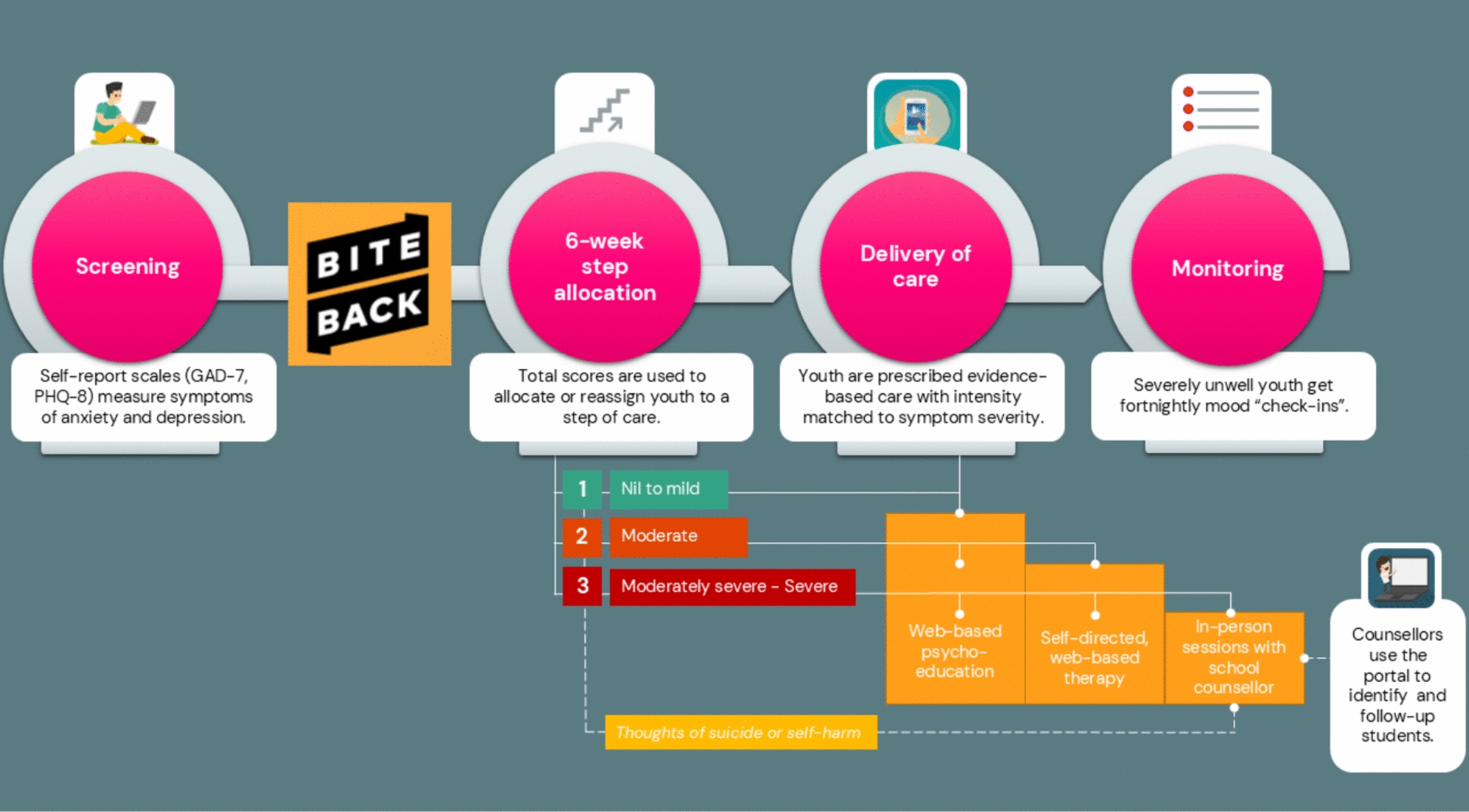
The revised *Smooth Sailing* service model: step criteria and care provided

The *Smooth Sailing* service is a universal, web-based mental health service for secondary school students developed by the Black Dog Institute [12, 27]. As shown in Fig. 1, the service screens students’ for symptoms of anxiety and depression using validated self-reported scales and then offers self-directed psychoeducation and internet-delivered Cognitive Behavioural Therapy (iCBT) modules for students with nil-to- moderate symptoms [28, 29], and in-person support from school counsellors for those with severe symptoms [12, 27]. A randomised controlled trial (RCT) demonstrated that the service significantly improved help-seeking intentions and reduced anxiety symptoms among secondary students [12], with minimal potential for harm [30]. However, rates of module completion were low. In the qualitative and quantitative feedback on the service, students reported time constraints, low motivation, and a perceived lack of need for care as key barriers to engagement, along with several suggestions for improvement [12].

To address these barriers, several modifications were made to the *Smooth Sailing* service, guided by the School Implementation Strategies Translating ERIC Resources (SISTER) framework [31] (see Supplementary Materials for the full range of strategies used, Table 1). To support engagement among asymptomatic students, a 6-week “watchful waiting” period was introduced, during which all participating students received the Bite Back Six-Week Challenge—a curriculum-aligned, positive psychology program [32, 33]. The student feedback process was also revised to include age-normed symptom reports with accompanying information on functional impacts and reasons for service engagement, along with a video explainer to increase understanding of the service. In addition, an evidence-based smartphone application was integrated to complement the web-based modules and enhance accessibility [34]. These enhancements were implemented uniformly across conditions to address barriers such as limited awareness and perceived irrelevance.

**Table 1:** Participant characteristics at baseline stratified by condition (N = 1295)

	SS (*n* = 469)		SS-CT (*n* = 335)		SS-FI (*n* = 491)		Total sample (N = 1295)	
	M	*SD*	M	*SD*	M	*SD*	M	*SD*
Age (years)	13.72	0.70	13.77	0.74	14.30	0.61	13.95	0.73
Help-seeking intentions (GHSQ)	33.55	10.74	33.58	10.44	34.14	9.76	33.79	10.30

	*n*	%	*n*	%	*n*	%	*n*	%
Female	232	49.5	121	36.1	290	59.1	643	49.7
Sexuality diverse^a^	42	9.0	25	7.5	31	6.3	98	7.6
Aboriginal or Torres Strait Islander	40	8.5	33	9.9	45	9.2	118	9.1
Previous or current mental health problem or diagnosis of a mental illness	129	27.5	106	31.6	107	21.8	342	26.4
Currently receiving psychological therapy	35	7.5	47	14.0	41	8.4	123	9.5
Currently taking medication for a mental illness	17	3.6	26	7.8	13	2.6	56	4.3
Previously or currently had a session with a school counsellor	122	26.0	92	27.5	138	28.1	352	27.2

^a^Identifies as either lesbian, gay, bisexual, transgender, queer, or intersex, LGBTQI

Control condition – Standard service = SS; Standard service + class time = SS-CT; Standard service + financial incentive = SS-FI.

Building on feedback from previous evaluations of the service and the broader literature, we also incorporated two additional uptake strategies for formal evaluation: (1) embedding rewards and incentives, and (2) providing students with dedicated class time for module completion [35]. These strategies were selected based on their alignment with the most commonly reported barriers—namely, time constraints and low motivation—and their feasibility within the school context. Importantly, these strategies could be systematically varied across school clusters, allowing a controlled evaluation of their impact on student engagement. Despite their promise, these strategies have not yet been systematically evaluated for effectiveness in enhancing uptake and adherence to digital mental health programs in school settings.

### Objectives of the current trial

This study examined the effectiveness of two student-level implementation strategies, class time allocation and financial incentives, on secondary school students’ uptake, engagement, and retention in the *Smooth Sailing* service. It was hypothesised that these strategies would increase student engagement, measured by the number of modules accessed, compared to the standard service. Service satisfaction and perceived barriers to use were also assessed. Additionally, this study explored the impact of these implementation strategies on the primary outcome of the service, help-seeking intentions for mental health problems. It was hypothesised that increased engagement would mediate improvements in help-seeking intentions.

## Methods

### Design

A three-arm, cluster randomised controlled trial (RCT) was conducted, with schools as clusters and students as individual participants. The reporting adheres to the CONSORT checklist for RCTs [36]. All outcome measures were assessed at the individual level. Data were collected at baseline, 6-weeks post-baseline, and 12-weeks post-baseline, with the primary outcome evaluated at 12-weeks post-baseline (primary endpoint). All participants reached the primary endpoint by December 2020. Ethical approval was granted by the University of New South Wales Human Research Ethics Committee (HC190382), the New South Wales Department of Education State Education Research Applications Process (2019302), the Catholic Schools Offices for the Dioceses of Maitland-Newcastle, Parramatta, and Wagga Wagga. The trial was registered with the Australian New Zealand Clinical Trials Registry (ACTRN12621000225819) and assigned the Universal Trial Number (U1111-1265-7440).

### Participants

The trial was conducted in secondary schools across South Australia (SA) and New South Wales (NSW), Australia. Schools were eligible to participate if they provided written Principal consent, had an onsite school counsellor or equivalent, and had reliable Internet access. All students in Grade 8 or Grade 9 with an active email address were eligible to participate unless their parent(s) or guardian(s) opted them out.

### Sample size

The target sample size was 1350 students (approximately 450 per condition), calculated to detect a small effect size (Cohen's d = 0.30) on a continuous primary outcome, with α = 0.5, and a 20% attrition rate. A conservative intraclass correlation (ICC) of 0.02 was assumed for possible clustering effects, slightly higher than the previous ICC of 0.017. Based on previous data [12], approximately 85 students per school were expected, requiring a minimum of 15 schools to meet the target sample size.

### Randomisation and blinding

Cluster randomisation at the school level was used to prevent contamination and for administrative feasibility [37]. Once principal consent was obtained, randomisation was performed by an external researcher not involved in trial operations , following International Council for Harmonisation (ICH) guidelines [38]. Schools were allocated in a 1:1 ratio using a minimisation approach [39, 40] to balance conditions by the Index of Community Socio-Educational Advantage (ICSEA) level (< 1000; > 1000), school type (co-educational; gender-selective), and grade level (Grade 8; Grade 9). Minimisation was conducted in Stata version 14.2 using the rct_minim procedure [41].

The initially available pool of schools was randomly sorted using Microsoft Excel random number generator, and schools were entered into the minimisation routine in ascending order based on the balancing variables. Schools that consented later were allocated using the same minimisation procedure once full information was provided. While the research program manager and one research officer were aware of the allocations due to administrative requirements, the broader research team remained blinded until the first school visit. Schools were informed of their assigned condition via email after providing consent. Students were not informed of their condition allocation at baseline. However, students in the financial incentive condition were notified at the 6-week mark—when the first incentive became available—and informed that continued incentives would be linked to their future engagement.

### Recruitment and consent

Schools were recruited via direct email invitations sent to relevant staff from the Black Dog Institute’s national school database. Additional recruitment efforts included advertisements in the Institute’s NSW School Counsellors e-Newsletter, the NSW School-Link newsletter (a government initiative that links schools with metropolitan and regional health services), the Black Dog Institute website, and social media platforms (Facebook and Twitter). Academic conferences, professional development courses, and state Departments of Education were also used to promote the trial.

Interested schools contacted the research program manager, who provided a study information package and followed up by phone. Once the school principal and school counsellor submitted a signed consent form, schools were allocated to their study condition, and the research team scheduled the school visits. Two weeks before the first visit, schools were instructed to distribute student information forms and a brief video about the service, with schools circulating service information through newsletters and other communication channels. Each participating school received a comprehensive implementation guide that outlined the service’s purpose along with clear, step-by-step instructions for setting up the service. This included guidance on school registration, forming a delivery team, selecting year groups, and promoting the service. The guide also described the screening process at 6- and 12-weeks, follow-up notifications for school counsellors, and how to use the School Counsellor Portal to ensure student safety. A supplementary guide was provided to School Counsellors with additional information about the School Counsellor Portal, and schools assigned to provide extra class time sessions received a supplementary guide with further details about arranging these  sessions.

An opt-out consent process was used, allowing parents to decline their child’s participation by notifying the school or research team before the first visit. In the Wagga Wagga Catholic Diocese, parental/guardian consent was required via an online or hardcopy form before the first visit. For all other students, informed consent was provided online on the day of the first assessment. All students participating completed a five-item Gillick competence test [42] to ensure student understanding of the study.

### Procedure

Implementation of the *Smooth Sailing* service was supported by three key teams. The *implementation team*, comprising research staff, coordinated service delivery, supported school personnel, developed the implementation guide, and tailored implementation approaches to individual school contexts. The *service team*, consisting of clinical and technical staff, managed IT infrastructure, enabled school access to the web-based platform, and produced support materials. *School liaisons*, drawn from the implementation team, served as primary points of contact for participating schools, facilitating communication, conducting site visits, and providing support throughout the service delivery and follow-up period. The specific implementation strategies delivered by these teams are outlined in Supplementary Material Table 1.

The study procedure was consistent across the three conditions, except that students in the class time condition received additional class periods to use the *Smooth Sailing* service, and students in the financial incentive condition received service reminders with tailored content. School liaisons conducted three assessment sessions during class time. Originally intended as face-to-face sessions, these were adapted to teleconferencing due to COVID-19, allowing schools to choose their preferred format.

At the first (baseline) assessment, students accessed the *Smooth Sailing* website via a school-specific Uniform Resource Locator (URL), provided online consent, registered by entering their name, gender, age, email, and mobile number (optional), and created a username and password. Mental health screening was conducted at baseline and repeated at 6- and 12-weeks post-baseline through a URL sent to students’ registered email (see Supplementary Material for more details).

After each assessment, school liaisons met with school counsellors, either in-person or online, to ensure they could access and navigate the web portal for student follow-ups. School counsellors received guides to interpret mental health scores and were instructed to follow school protocols for student support, referrals, and parental contact when needed. They were also given a list of local mental health services. School liaisons used standard instruction sheets for these meetings and did not provide clinical supervision. Three days after each school visit, researchers reviewed the School Counsellor Portal to confirm all follow-ups were completed and monitored for adverse events. If needed, they contacted school counsellors to update the portal accordingly.

### Implementation strategies and conditions

#### Control condition—*Standard service (SS)*

Schools and students received the standard *Smooth Sailing* service. Screening activities occurred during class and students completed the service modules independently, in their own time.

#### Standard service + class time (SS-CT)

In addition to the standard service, students in this condition received two extra class periods to access the service and complete modules. There were no restrictions on the type or duration of the class periods; however, most Australian secondary school class periods range from 45–75 min.

#### Standard service + financial incentive (SS-FI)

In addition to the standard service, students in this condition were informed at the 6-week mark that they would receive 3.0 Australian Dollars (AUD) for each of the first five modules completed and 2.5AUD for each of the 6- and 12-week screening assessments, up to a maximum reimbursement of 20.0AUD. The incentive amount was selected to balance feasibility and scalability within school-based settings, offering a modest reward to encourage engagement while maintaining ethical considerations. The amount was based on the minimum wage for a 16-year-old in Australia at the time of the study (11.40AUD per hour), equivalent to approximately 0.20AUD per minute.

### Outcome measures

#### Service engagement

The primary outcome was the number of modules accessed by students. The *Smooth Sailing* service included seven modules: Bite Back Six-Week Challenge, five psycho-education modules, and one iCBT module. Engagement was measured as the proportion of modules accessed, which varied depending on each student’s step allocation at the 6- and 12-week time points.

#### Service uptake

Uptake was defined as the number of students who registered for the service at baseline.

#### Service retention

Retention was defined as the proportion of registered students who completed the 12-week mental health screening assessment.

#### Help-seeking intentions for mental health problems

Students completed the General Help-Seeking Questionnaire (GHSQ: [43]) at baseline, 6- and 12-weeks post-baseline to assess the likelihood of seeking help for mental health problems. Items were rated on a 5-point Likert scale from “extremely unlikely (1)” to “extremely likely (5)” (α = 0.89). Higher scores indicated a greater likelihood to seek help across 13 sources, with an option to specify additional sources. The GHSQ is widely used among adolescents aged 11–18 years and has shown satisfactory psychometric reliability [44].

#### Service satisfaction and barriers to use

At 12 weeks post-baseline, students completed an 11-item questionnaire assessing satisfaction with the service, including perceptions of enjoyment, ease of use, usefulness, and comfort. Items were rated on a 5-point Likert scale, and students also provided an overall rating of the service’s helpfulness on the same scale. An additional 18-item questionnaire assessed perceived barriers to service use, covering technical, personal, and intervention-specific factors. Responses were dichotomous (yes/no).

#### Demographics and mental health history

Students reported their age, gender, sexual identification, and Aboriginal and Torres Strait Islander status. They were asked whether they had ever experienced or been diagnosed with a mental health illness such as depression or anxiety (yes, no, I’d rather not say). Additionally, students indicated if they were currently receiving treatment or taking prescribed medication for a mental health condition using the same response options. A separate question asked whether they had ever attended a session with the school counsellor (yes, no, I’d rather not say).

### Data collection and analysis

Data were securely collected and stored using the Black Dog Institute research engine hosted on the University of New South Wales servers. Data was extracted using Microsoft Excel and analysed using Statistical Package for the Social Sciences (SPSS) version 27.0 (SPSS Inc, Chicago, I1, USA, 2017). Analyses followed an intention-to-treat approach, including all randomised participants regardless of the intervention received.

To examine module access, a series of models for count data were fitted to the number of modules accessed by each participant, with group assignment included as a fixed factor and school as a random factor to account for clustering effects. Based on the 6- and 12-week screening assessments following Bite Back completion, the maximum number of available modules per participant was included as an exposure offset variable. The cluster-specific incident rate ratio (IRR) was used to compare module completion rates between each enhanced condition and the standard service for an ‘average’ school (i.e., a school with a random effect of 0). Individual school effects and the model’s overdispersion parameter were considered.

To evaluate the population-level impact of each intervention on module access in the full sample, the intraclass correlation coefficient (ICC) was estimated using the trigamma function to assess observation-level variance, including the maximum number of modules as a fixed effect [45]. Given the limitations of count and logistic models in accommodating the increased proportion of participants accessing more modules at the upper distribution, alternative model formulations such as the proportional odds model were explored. This model assumes constant odds of transitioning between modules regardless of the number of modules accessed.

Uptake and retention rates were analysed using mixed-effects logistic regression, with school included as a random effect to accommodate for clustering effects. The effects of the implementation strategies on help-seeking intentions were assessed using linear mixed-effects models, comparing changes over time and differences between conditions. A sub-group analysis was also conducted to explore the relationship between service engagement and help-seeking intentions.

## Results

### Sample

**Fig. 2:**
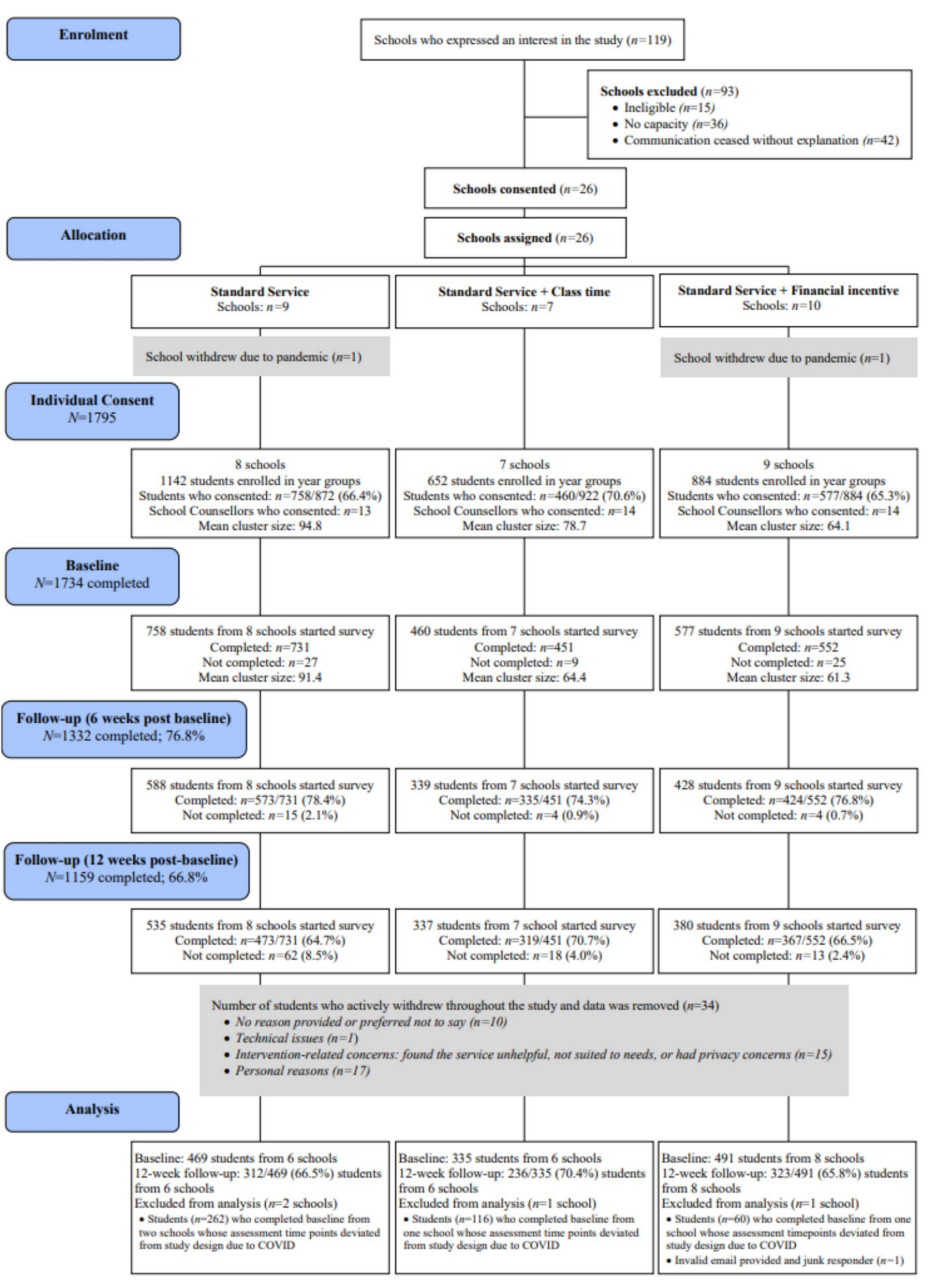
Consort diagram showing participation, withdrawals and attrition

As shown in Fig. 2, 119 schools expressed interest in the study, and 26 (21.9%) agreed to participate. Nine schools were allocated to the standard service condition (*n* = 5, 62.5% located in major cities), 7 to the class time condition (*n* = 3, 42.9% located in major cities), and 10 were allocated to the incentive condition (*n* = 6, 60% located in major cities). Two schools withdrew due to COVID-19 after allocation but before the baseline assessment.

In total, 1795 students from 24 schools consented and completed the baseline assessment. However, due to COVID-19 closures, four schools that commenced participation in Term 1 (between 11th February 2020 and 19th March 2020), experienced interruptions and later resumed data collection between 23rd July 2020 and 5th August 2020, with the final school visits occurring in Term 3 (3rd September 2020 to 17th September 2020). The remaining 20 schools commenced the trial in Term 3, 2020 (10th August 2020 to 17th September 2020), and completed the trial in Term 4 2020 (2nd November 2020 to 10th December 2020). The four schools (*n* = 438) that started in Term 1 were excluded from the analyses due to the extended delay between the first two study assessments. The final sample consisted of 1295 students from 20 schools.

In the final sample, approximately half of the participants were female (*n* = 643/1295; 49.7%), with a mean age of 13.95 years (SD: 0.73; range 12–16 years). One in four students reported a past or current mental health problem (*n* = 342/1295, 26.4%), 9.3% (*n* = 123/1295) were receiving psychological therapy, and 27.2% (*n* = 352/1295) had previously had a session or were currently seeing their school counsellor. Participant characteristics are summarised in Table 1.

### Service engagement: modules accessed

Table 2 shows the number of students who accessed Bite Back and each module in the service.

**Table 2:** Total number of modules accessed by the total sample and stratified by condition (N = 1295)

	SS (*n* = 469)		SS-CT (n = 335)		SS-FI (*n* = 491)		Total sample (N = 1295)	
	*n*	%	*n*	%	*n*	%	*n*	%
Bite back	231	49.3	259	77.3	284	57.8	774	59.8
Module 1	80	17.1	81	24.2	162	33.0	323	24.9
Module 2	161	34.3	165	49.3	226	46.0	552	42.6
Module 3	86	18.3	79	23.6	152	31.0	317	24.5
Module 4	60	12.8	61	18.2	134	27.3	255	19.7
Module 5	76	16.2	71	21.2	142	28.9	289	22.3
Module 6^a^	50	29.4	58	45.7	96	50.3	204	41.8

^a^Module 6 was only available to participants at Steps 2 + so the percentage was calculated by dividing the number of students who accessed this module by the number of users allocated to Steps 2 + at 6- or 12-weeks (*n* = 170, 127, and 191 respectively)

**Fig. 3:**
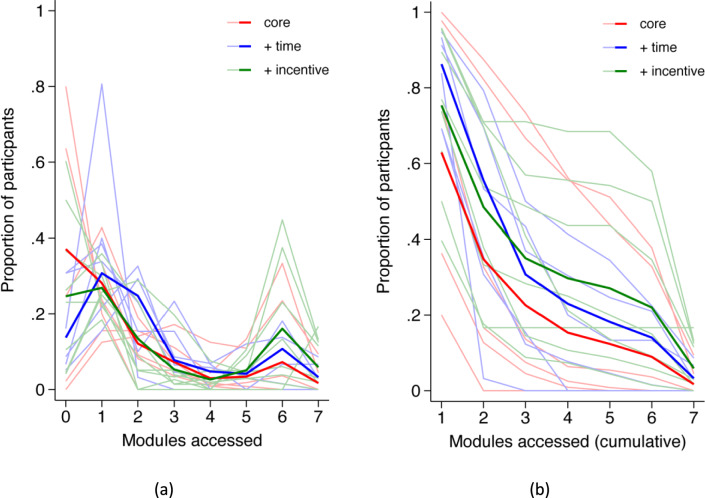
**a** Observed proportion and **b** cumulative proportion of modules accessed for each school (thin lines) and mean proportions (thick lines) for each condition.

As shown in Fig. 3, a considerable proportion of participants across all conditions did not access any modules or progress beyond the first module. Rates of module access also varied substantially between schools. Across all schools, 46.7% of students (*n* = 605/1295) did not access Bite Back or any other modules. Over half of the students (54.8%, *n* = 257/469) allocated to the standard service did not access any modules, including Bite Back. Among students allocated to the class time condition, 38.8% (*n* = 130/335) of students did not access any modules, while 44.4% (*n* = 218/491) of students in the financial incentive condition did not access Bite Back or any other modules.

Table 3 shows the estimated mean count for each condition. While each implementation strategy increased the mean number of modules accessed, the improvement from the standard service was not statistically significant in either case (*p* = 0.14).

**Table 3:** Estimated marginal mean number of modules accessed for each intervention and differences from standard service for the full sample

Condition	Estimated count (95% CI)	Comparison with standard service (SS)	
		Estimated difference (95% CI)	p value
Standard service (SS)	1.53 (0.63–2.42)	–	–
Standard service (SS) + class time (SS-CT)	2.21 (0.91–3.52)	0.68 (− 0.86 to 2.22)	0.39
Standard service (SS) + incentive (SS-FI)	2.76 (1.33–4.18)	1.23 (− 0.40 to 2.86)	0.14

A mixed effects negative binomial model fitted the data substantially better than a Poisson model (χ^2^ = 118.21, df = 1, *p* < 0.001), and better than a negative binomial model omitting the random effect of school (χ^2^ = 409.13, df = 1, *p* < 0.001). Comparison of observed and expected counts and examination of Pearson residuals showed this model fitted the highly prevalent low modules accessed end of the data distribution extremely well, with the only source of poor fit attributable to high module access (6 and 7 modules), notably in the incentive condition. For the class time condition, the IRR of 1.45 (95% CI: 0.64–3.25) was not significant (z = 0.90, *p* = 0.37). Similarly, the IRR of 1.80 (95% CI: 0.85–3.84) for the incentive condition was not significant* z* = 1.53, *p* = 0.13).

### Uptake and service retention

Figure 2 displays the uptake rates across the three conditions. Uptake rates for the standard service, class time, and financial incentive conditions for the final sample of students across the 20 schools were 59.1% (*n* = 492/832), 65.1% (*n* = 343/527), and 65.6% (*n* = 514/784), respectively, with no significant differences between them (χ^2^ = 1.18, df = 2, *p* = 0.56). Retention rates at 12-weeks post-baseline were 66.5% (*n* = 312/469) for the standard service, 70.4% (*n* = 236/335) for class time, and 65.8% (*n* = 323/491) for the financial incentive condition , also showing no significant differences (χ^2^ = 0.11, df = 2, *p* = 0.95).

### Effects of modules accessed on help-seeking intentions

**Fig. 4:**
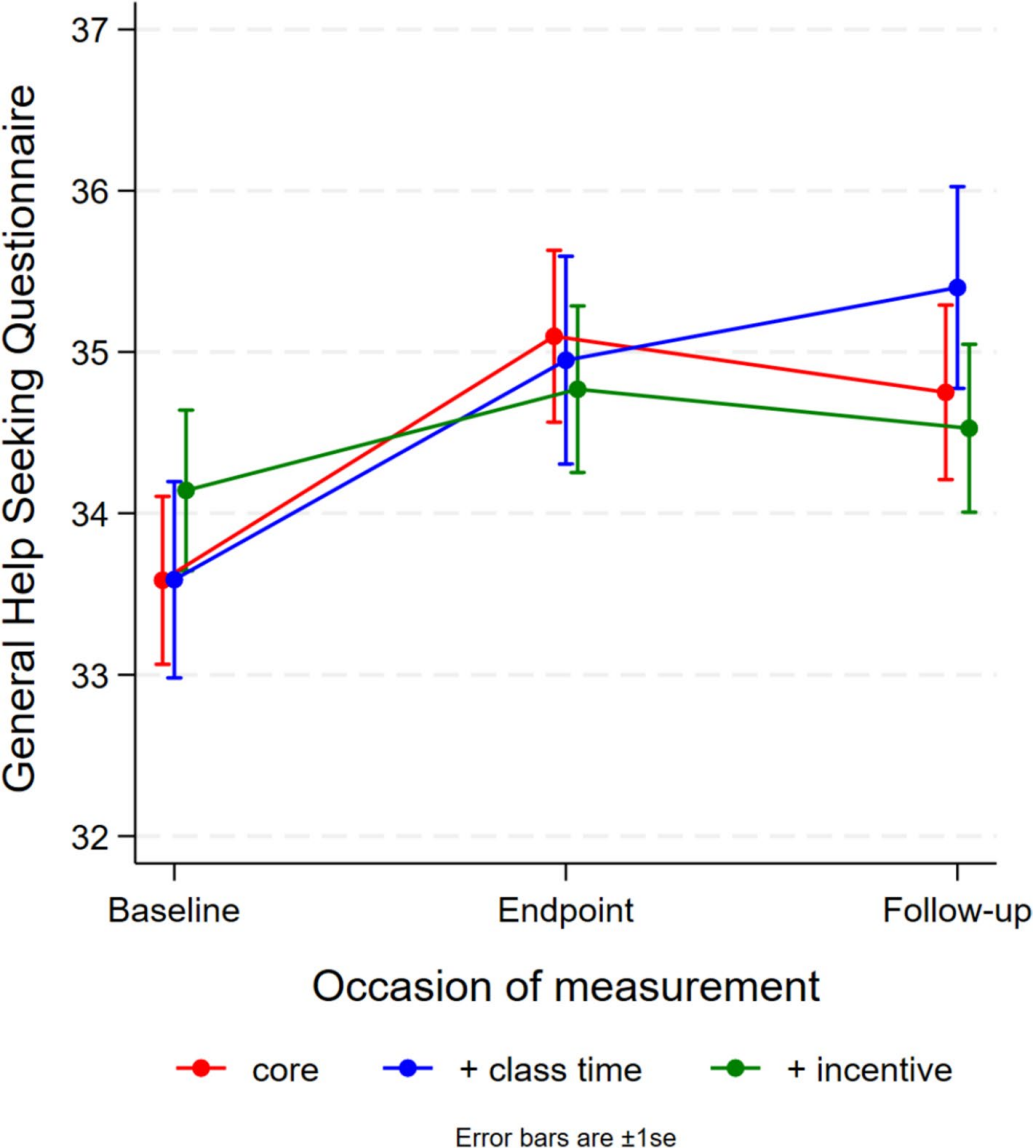
Help-seeking intentions by condition and occasion of measurement

There were no significant differences in help-seeking intentions between conditions at any time point. However, as shown in Fig. 4, both the standard service and class time conditions demonstrated significant improvements from baseline to 6-weeks post-baseline, with mean increases of 1.51 (*t* = 3.30, df = 1001.1, *p* = 0.001) for the standard service and 1.36 (*t* = 2.42, df = 1115.2, *p* = 0.02) for the class time condition. Similarly, significant improvements were observed at 12-weeks post-baseline, with mean increases of 1.16 (*t* = 2.34, df = 14.15.7, *p* = 0.02) for the standard service and 1.81 (*t* = 3.14, df = 1281.7, *p* = 0.002) for the class time condition. No significant changes were observed for the incentive condition at either time point (all P > 0.05).

**Fig. 5:**
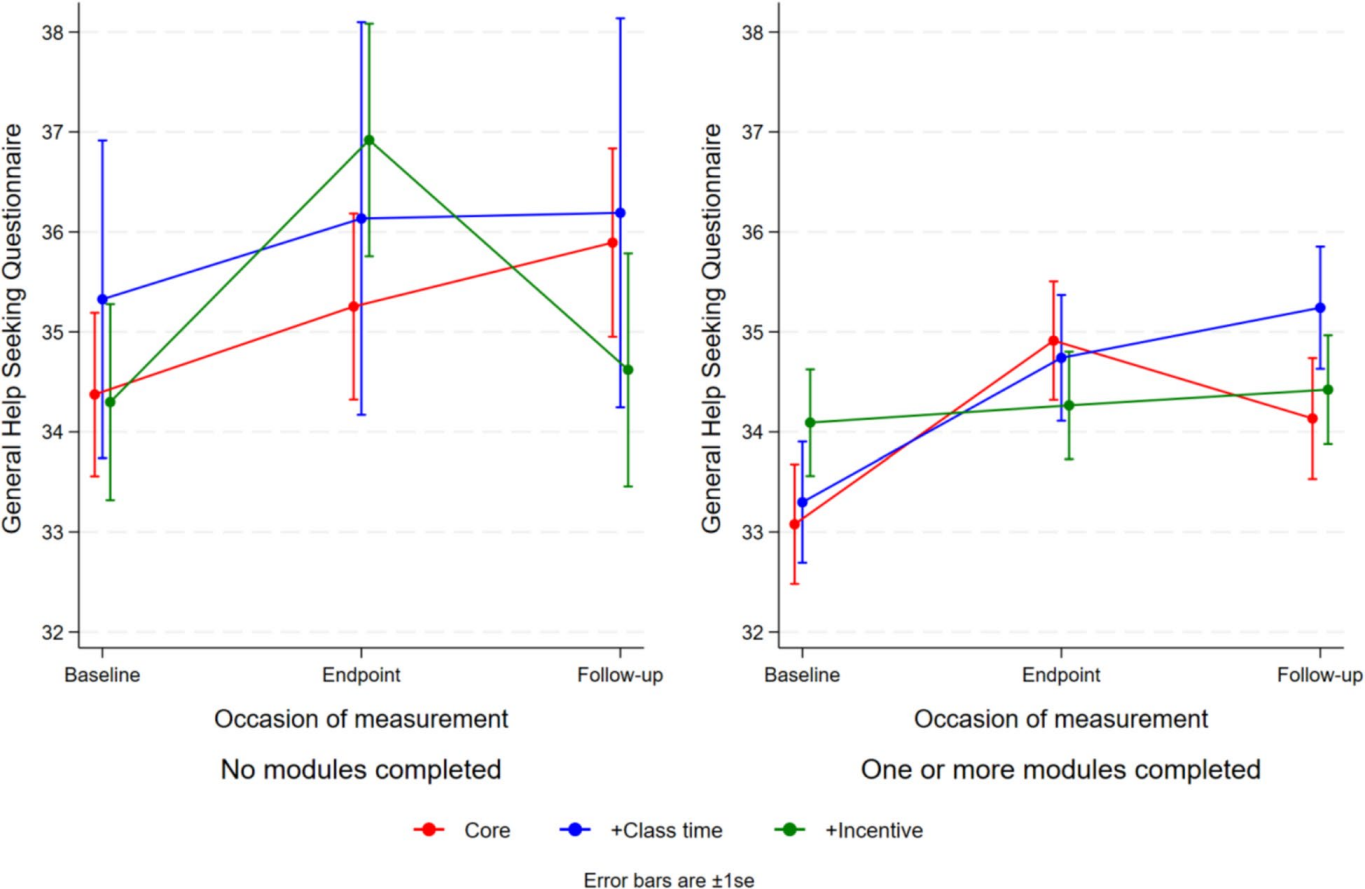
Help-seeking intentions by condition and occasion of measurement for students who accessed **a** no modules, and **b** one or more modules

To explore the relationship between service engagement and help-seeking intentions, a categorisation of “no modules” versus “one or more modules” accessed was used. There were some significant differences in each category. As shown in Fig. 5, among students who completed no modules, those in the incentive condition demonstrated significantly smaller improvements in help-seeking intentions from baseline compared to students receiving the standard service. Conversely, students in the standard service and class time conditions showed greater improvement, while students in the incentive condition showed minimal change. Among students who completed one or more modules, those in the incentive condition maintained relatively stable help-seeking intentions, initially starting slightly higher than those in the standard service and class time conditions. However, students allocated to receive the standard service and who were allocated additional class time caught up to varying extents.

### Service satisfaction and barriers to use

As shown in Table 4, across all conditions, most students found the service easy to use and understand, and over half found the service interesting and enjoyable. Almost two-thirds of all students were comfortable with the school counsellor follow-ups. Across all conditions, less than half indicated that they would use the service again or found the service helpful in managing their daily lives or their feelings. Students across all conditions cited personal barriers such as forgetfulness, low motivation, and service mismatch with needs as barriers to service use. Intervention-specific and technical barriers were less prevalent than personal barriers across all conditions, with a quarter or fewer students citing these issues as impeding their use of the service. Mean helpfulness ratings were 3.21 (SD: 1.00) for the standard service, 2.89 (SD: 1.03) for the class time condition, and 3.19 (SD: 0.99) for the financial incentive condition (range: 1 to 5).

**Table 4:** Service satisfaction and barriers to use at 12 weeks post-baseline

	SS (*n* = 315)		SS-CT (*n* = 246)		SS-FI (*n* = 329)	
Satisfaction	*n*	%	*n*	%	*n*	%
Easy to use	286	90.8	207	84.1	295	89.7
Easy to understand	278	88.3	209	85.0	288	87.5
Interesting to use	193	61.3	126	51.2	213	64.7
Enjoyed using	190	60.3	132	53.7	205	62.3
Would recommend it to a friend	194	61.6	126	51.2	214	65.0
Would use it again in the future	126	40.0	90	36.6	150	45.6
Helped me to feel in control of my feelings	127	40.3	87	35.4	158	48.0
Skills I leaned helped me in everyday life	111	35.2	61	24.8	143	43.5
I was comfortable with being followed up by school counsellor if required	194	61.6	152	61.8	210	63.8
I was comfortable proving my email address	220	69.8	155	63.0	227	69.0
I was comfortable providing my mobile number	133	42.2	83	33.7	157	47.7

	SS (*n* = 315)		SS-CT *(n* = 245)		SS-FI (*n* = 329)	
Barriers to use	*n*	%	*n*	%	*n*	%
Forgetfulness	149	47.3	115	46.9	169	51.4
Low motivation	131	41.6	123	50.2	156	47.4
Felt it wasn’t what was needed	126	40.0	99	40.4	140	42.6
Lack of time	85	27.0	93	38.0	139	42.2
Didn’t want school counsellor to know my feelings	76	24.1	58	23.7	73	22.2
Data privacy concerns	58	18.4	43	17.6	68	20.7
Felt to worried or too down	29	9.2	23	9.4	32	9.7
Took too long to read	60	19.0	62	25.3	60	18.2
Made me feel worse	46	14.6	50	20.4	45	13.7
Used too much phone data	26	8.3	26	10.6	31	9.4
Too hard to read on phone	19	6.0	34	13.9	28	8.5
Couldn’t complete activities on phone	35	11.1	30	12.2	38	11.6
Forgot how to access	39	12.4	42	17.1	46	14.0
Faulty internet connection	27	8.6	38	15.5	56	17.0
Difficulty logging into website	24	7.6	30	12.2	24	7.3
Took too long to load	27	8.6	32	13.1	30	9.1
Didn’t have a smartphone or computer	9	2.9	11	4.5	9	2.7

For satisfaction and barriers to use, *n* are participants who completed the final follow-up survey and agreed with each statement

## Discussion

This trial represents one of the few attempts to formally evaluate the effectiveness of implementation strategies aimed at increasing student engagement and uptake of a universal school-based digital mental health intervention. Overall, student engagement in the intervention remained low across all three conditions, despite the use of targeted implementation strategies. The evaluated strategies did not lead to significant differences in uptake or retention among students. Although prior research has demonstrated some evidence supporting the use of these strategies for improving individuals’ engagement with interventions in structured settings (e.g., 20) and for increasing retention and participation (e.g., 21, 23, 25), the use of such strategies to supporting the implementation of digital mental health interventions in Australian secondary schools requires further refinement.

Contrary to the hypotheses, students who received financial incentives were not more likely to engage with the service. Students who received financial incentives also showed smaller improvements in help-seeking intentions compared to students who received the standard service or additional class time, particularly if they did not complete any modules. In this trial, the use of external rewards may have undermined intrinsic motivation, particularly once the incentive was removed [46]. Prior literature emphasises the importance of intrinsic motivation for sustaining engagement in interventions [47], and highlights the challenges of relying on extrinsic motivators, like financial rewards, for lasting behaviour change [48]. For example, a recent study examining motivation in an 8-week self-guided web-based intervention found a positive correlation between internal motivation and intervention initiation, adherence, and satisfaction [49]. Students with moderate to high internal motivation also exhibited greater symptom improvement. Thus, future research could explore strategies to enhance intrinsic motivation by incorporating elements that foster autonomy (e.g., personalisation of goals and activities, flexible scheduling) and competence (e.g., skill-building exercises, progress tracking) to improve engagement and outcomes in digital interventions. In contrast, studies conducted in paid research contexts have shown higher intervention uptake and completion rates among young people compared to naturalistic settings (e.g., 24). However, the modest incentive offered in this study may have been insufficient to meaningfully influence engagement. Future research could examine which types and magnitudes of feasible extrinsic incentives provide the greatest value for enhancing engagement among school-based youth.

Service quality improves when program goals, rationale, and components are clearly communicated, feedback on program outcomes is provided, implementation barriers are addressed with well-developed plans, and individual responsibilities are well-defined [7, 50]. Although participating schools received a comprehensive implementation guide detailing the purpose and benefits of the service, step-by-step delivery process, and regular support via emails, telephone calls, and checklists, module access varied widely across schools. Some schools demonstrated high levels of engagement across students, while others had many students who did not access any modules. This variation suggests that other contextual factors may have influenced engagement. Participating schools included government, non-government and special assistance schools, the latter offering flexible learning environments for students who struggle in traditional settings. Schools also differed by location (rural, regional, metropolitan), affecting class structure, attendance, and teacher support. Government schools, for example, generally havehigher student-to-staff ratios than non-government schools (including Catholic and independent schools), and rural government schools often face additional challenges such as staff shortages, higher turnover, and limited access to mental health resources such as counsellors and psychologists [51, 52]. Future research involving a larger number of schools could examine which school characteristics predict higher engagement with digital mental health interventions. In addition, tailoring implementation strategies to the unique context of each school and the specific roles of its staff (e.g., [9, 53, 54]) may improve feasibility and engagement compared to a one-size-fits-all approach focused solely on student-level factors. However, this approach introduces challenges, including maintaining implementation consistency, accurately assessing fidelity, and clearly defining the intervention across diverse settings. Such variability may complicate the interpretation of outcomes, reduce internal validity, and limit the ability to attribute effects to specific intervention components. Future trials should carefully balance these trade-offs and consider mixed-method approaches to better understand how contextual adaptations influence service implementation and outcomes.

Importantly, the COVID-19 pandemic introduced significant challenges, with teachers and schools facing additional disruptions and stressors during this time. During the pandemic, teachers reported heightened stress, exhaustion, and low positivity in their work due to the demands of remote learning and increased responsibilities from students, parents, and school leaders [55]. Many schools in this study shifted the service delivery from in-person to online formats to comply with government guidelines and school preferences, which affected how the service was implemented. During these virtual sessions, school liaisons were present on-screen, but student environments varied—some students were placed in smaller classrooms, while others were seated in larger halls or theatres. Remote, online delivery worked well when staff were organised, provided adequate supervision, and actively engaged students. However, module completion rates were likely impacted by inconsistent or low supervision, less engaged staff, and varying adherence to service requirements in less structured settings.

Additionally, many students may have been overwhelmed by competing demands and stressors during the pandemic, contributing to the low engagement with the modules. Nearly half of the students across all conditions cited forgetfulness, low motivation, and a perception that the service was not what they needed as the main barriers to using the service. These barriers, such as time constraints and low motivation, mirror those identified in prior evaluations of the service [12] and other similar studies [8, 56]. The additional engagement measures, such as providing a short information video about the service and incorporating more comprehensive feedback following screening, may have been insufficient to meaningfully boost student engagement. Alternatively, students may not have found the service relevant at the time, particularly in the context of heightened loneliness, isolation, and uncertainty during the pandemic (e.g., [57]). While the service offered some personalisation and choice, factors known to boost motivation and engagement with digital mental health interventions [58], future studies could explore alternative or complementary strategies such as moderated peer-support groups or platforms for students to share experiences (e.g., [15, 59]) and approaches that explicitly target intrinsic motivation (e.g., [49]). Notably, 15.9% of participants (n = 141/889) in this study reported that the service "made them feel worse" at the primary endpoint. This contrasts with earlier evaluations of the *Smooth Sailing* service, where the barrier did not exceed the benchmark for service modification (i.e., > 20% endorsement) [27], with raw values indicating that no participants reported feeling worse in the pilot. In the subsequent RCT, only 7.2% of participants reported this experience [12]. This discrepancy warrants further investigation. Furthermore, our additional harms analysis [30] found no overall increase in mental health-related harms associated with more intensive screening procedures, and instead showed evidence of improved help-seeking behaviour. Given the absence of formally reported adverse events and the low rates of module completion, these findings highlight the need to explore factors contributing to negative perceptions— whether related to specific content, delivery mechanisms, or aspects of the research process. 

There is growing recognition of the potential for iatrogenic effects in universal school-based mental health programs, particularly when implemented at scale without sufficient contextual adaptation [60–63]. This study contributes to the emerging literature urging careful evaluation of unintended consequences of universal approaches, including distinguishing harms arising from intervention content or delivery from those linked to research participation. As Cohen et al. note, many universal interventions are implemented in isolation from the broader school ecology, without integration into multitiered systems of support or sufficient student voice and autonomy—factors that can undermine both engagement and effectiveness [63]. To address these concerns, future digital school-based interventions should adopt participatory co-design approaches that meaningfully involve students in shaping both content and delivery [63–65]. Strengthening implementation strategies and ensuring flexibility to accommodate students’ preferences and developmental needs may improve effectiveness and reduce the risk of unintended negative outcomes [64].

### Limitations

The findings should be viewed considering several limitations. First, the impact of COVID-19 on data collection may have influenced student engagement, as disruptions to normal school operations and pandemic-related stressors could confound the study results. Second, the final sample consisted of students from 20 out of 119 initially interested schools, with two schools withdrawing due to the pandemic. This non-random attrition introduces potential selection bias, which could affect the generalisability of the results. Geographical and contextual limitations also apply, as most participating schools were from NSW and one independent school from SA, limiting generalisabilityto other states or territories in Australia or countries with different cultural or educational contexts. Additionally, participation depended on principal approval and ethical clearance, which excluded some Catholic Dioceses within NSW and outside of NSW and may have prevented participation from interested staff in other schools.

 At the school level, effective implementation requires progress monitoring, feedback, school personnel buy-in, and regular meetings [66, 67]. Although staff received regular support and communication to review processes and track progress, pandemic-related disruptions redirected efforts and resources toward rescheduling school visits, developing additional protocols, and supporting schools for online service delivery. As a result, no formal data on adherence to the implementation protocol were collected. This lack of adherence data may limit our understanding of how consistently the program was delivered across schools. Ensuring and safeguarding program fidelity should be a key focus for future research. Additionally, although schools were provided with a comprehensive and user-friendly implementation guide, its length and static PDF format may have presented a time burden—particularly for schools with limited staffing or competing priorities. Future iterations of the guide may benefit from co-design with educators to streamline content and improve accessibility. Incorporating more dynamic formats, such as interactive digital tools or brief instructional videos, could enhance usability and support engagement across diverse school settings.

## Conclusion

The strategies of providing extra class time and financial incentives did not significantly improve student engagement or help-seeking outcomes. Variability in school environments, staff involvement, and external stressors—particularly those related to the COVID-19 pandemic—likely contributed to the inconsistent levels of student engagement across schools. These findings highlight the importance of considering the broader school context when implementing mental health services. Future efforts should prioritise implementation strategies that are not only flexible and tailored to the school setting but also informed by meaningful student involvement. Participatory co-design approaches and alternative delivery formats may help improve engagement, relevance, and outcomes, particularly for digital mental health programs delivered in school settings.

## Supplementary Information

Supplementary material 1.

## Data Availability

The datasets generated and analysed during the current study are not publicly available due to ethical and data-sharing restrictions. However, data may be available from the corresponding author upon reasonable request, subject to institutional ethics approval and data-sharing agreements.

## Abbreviations

AUD: Australian Dollar
GHSQ: General Help-Seeking Questionnaire
iCBT: Internet-delivered Cognitive Behavioural Therapy
ICC: Intraclass correlation
ICH: International Council for Harmonisation (ICH)
ICSEA: Index of Community Socio-Educational Advantage (ICSEA)
IRR: Incident rate ratio (IRR)
NSW: New South Wales
RCT: Randomised Controlled Trial
SA: South Australia
SISTER: School Implementation Strategies Translating ERIC Resources
SPSS: Statistical Package for the Social Sciences
SS: Standard service
SS-CT: Standard service + class time
SS-FI: Standard service + financial incentive
URL: Uniform Resource Locator
